# Does contact with the justice system deter or promote future delinquency? Results from a longitudinal study of British adolescent twins†

**DOI:** 10.1111/1745-9125.12236

**Published:** 2019-12-29

**Authors:** Ryan T. Motz, J.C. Barnes, Avshalom Caspi, Louise Arseneault, Francis T. Cullen, Renate Houts, Jasmin Wertz, Terrie E. Moffitt

**Affiliations:** ^1^ School of Criminal Justice University of Cincinnati; ^2^ Department of Psychology & Neuroscience Duke University; ^3^ Department of Psychiatry & Behavioral Sciences Duke University School of Medicine; ^4^ Center for Genomic and Computational Biology Duke University; ^5^ MRC Social, Genetic, & Developmental Psychiatry Centre, Institute of Psychiatry, Psychology, & Neuroscience King's College London

**Keywords:** delinquency, family fixed effects, labeling, specific deterrence, twins

## Abstract

What impact does formal punishment have on antisocial conduct—does it deter or promote it? The findings from a long line of research on the labeling tradition indicate formal punishments have the opposite‐of‐intended consequence of promoting future misbehavior. In another body of work, the results show support for deterrence‐based hypotheses that punishment deters future misbehavior. So, which is it? We draw on a nationally representative sample of British adolescent twins from the Environmental Risk (E‐Risk) Longitudinal Twin Study to perform a robust test of the deterrence versus labeling question. We leverage a powerful research design in which twins can serve as the counterfactual for their co‐twin, thereby ruling out many sources of confounding that have likely impacted prior studies. The pattern of findings provides support for labeling theory, showing that contact with the justice system—through spending a night in jail/prison, being issued an anti‐social behaviour order (ASBO), or having an official record—promotes delinquency. We conclude by discussing the impact these findings may have on criminologists’ and practitioners’ perspective on the role of the juvenile justice system in society.

The juvenile justice systems of the Western world were established to embrace the doctrine of *parens patriae* and to emphasize the treatment and rehabilitation of wayward youth (Parsloe, 1978). Yet, as the world was swept by dramatic social change in the 1960s and 1970s, many began to question the legitimacy of social institutions like the justice system. Part of this skepticism was from a relative lack of faith in correctional programming, which was partially driven by two damning critiques—one from the United States (Martinson, 1974) and one from the United Kingdom (Brody, 1976)—of its ability to rehabilitate offenders and reduce recidivism. In particular, after reviewing the available evidence, Martinson (1974, p. 25) concluded, “[T]he rehabilitative efforts that have been reported so far have had no appreciable effect on recidivism.” Brody (1976, p. 37) similarly stated, “Reviewers of research … have unanimously agreed that the results have so far offered little hope that a reliable and simple remedy for recidivism can be easily found.” Sweeping changes followed both reports. For example, there were reductions in rehabilitative programming for both juvenile delinquents and adult offenders in their respective countries, which ultimately led to the “get tough” era in the United States (Cullen & Gilbert, 2013) and the implementation of the neo‐correctionalist model in the United Kingdom (Cavadino & Dignan, 2006). Motivated by fear of the “super‐predator” (Bennett, DiIulio, & Walters, 1996), these movements opened the door for the philosophies of deterrence and incapacitation to form the basis for many policy decisions.

But the pendulum seems to be swinging back toward rehabilitation in recent years as both the United States and the United Kingdom have reduced their use of punitive actions against delinquent youth (Bateman, 2017; Taylor, 2017). Such trends are at least partially the result of a movement away from conservative era policies because of an increase in the discomfort with deterrence theory and the revival of a fear that labeling effects are indeed real. These shifts in policy have shone light on one of the classic theoretical debates in criminology: Does contact with the justice system deter or promote future criminal behavior? Two long‐standing theoretical traditions—deterrence theory and labeling theory—have been focused on answering this question, but they have arrived at different conclusions. Both have shifted the primary focus away from the individual offender and have placed it on the actions and impact of the justice system where contact with the system works as a turning point that alters the life courses of individuals (Elder, 1985; Laub & Sampson, 1993; Sampson & Laub, 2003). According to deterrence theory, justice system contact is a positive turning point, implying contact reduces future offending by teaching offenders the costs of crime outweigh the benefits. According to labeling theory, justice system contact is a negative turning point, implying contact exacerbates the chances of later offending by initiating a self‐fulfilling prophecy in which the individual perceives him‐ or herself as a “bad apple.”

Although deterrence and labeling have rich theoretical histories and long empirical records (see Farrington & Murray, 2014; Nagin, Cullen, & Jonson, 2018), few studies have been designed to adjudicate between the two frameworks in a broadly generalizable way. There have been many tests of deterrence theory and labeling theory independently, but few researchers have been able to sort out which theory offers the best explanation of the impact of typical justice system contact. The ones that sought to do so have had mixed results (e.g., Klein, 1974; Nedelec & Silver, 2018; Smith & Gartin, 1989; Ward & Tittle, 1993; Wiley & Esbensen, 2016), leaving the difficult task of having to draw on an inconclusive evidence base when making policy recommendations.

One factor partially responsible for the mixed evidence base is that much of the available research has had methodological designs that may not adequately account for preexisting differences between individuals that affect variation in their risk of engaging in offending behavior (see Loughran, Paternoster, & Piquero, 2018). In particular, scholars have sought to test deterrence and labeling, but they have tended to do so with research designs that cannot disentangle causal effects from selection effects as a result of their reliance on observable variables to control for selection. Consequently, sources of unobserved selection bias may confound estimates of the causal impact of justice system contact on subsequent antisocial behavior, complicating the interpretation of prior results (Jacobs, 2010; Morris & Piquero, 2013; Morris, Barnes, Worrall, & Orrick, 2013).

In this study, we test the relationship between common forms of justice system contact in the United Kingdom and later delinquency to offer insight into the long‐standing “deterrence or labeling” question. We account for the confounding influences of selection bias by leveraging a unique sample and a powerful methodological design that has been recognized for its ability to rule out a wide range of biasing influences: a family fixed‐effects design. The family fixed‐effects design has appeared in the literature under other names such as the “co‐relative approach” (Kendler, 2017), “discordant twin design” (Moffitt & Beckley, 2015), “twin difference approach” (Nedelec & Silver, 2018), and “sibling‐comparison analysis” (Connolly & Kavish, 2018). Regardless of the name, the design is implemented with the goal of adjusting for a wide range of potential confounds at the family level, factors such as the early rearing environment, neighborhood effects, and even genetic inheritance (Becker & Tomes, 1986; Heckman & Mosso, 2014). Essentially, family confounds capture all factors that might operate to make members of the same family similar to one another. As a result, the design offers “a natural solution” to ruling out many sources of confounding in criminological research (Moffitt & Beckley, 2015, p. 123).

We draw on data from a nationally representative and longitudinal study of British twins to estimate the effect of coming into contact with the justice system on subsequent delinquency. In particular, the effect of the following three common forms of justice system contact are explored: 1) spending a night in jail/prison, 2) being labeled by the justice system through what was known as an anti‐social behaviour order (ASBO), and 3) having an official crime record. Aside from the United Kingdom having a much lower incarceration rate, its justice systems are comparable with those of the United States. The age of criminal responsibility is 10 years, and 18 years is the age that separates the juvenile system from the adult system.

Given that the ASBO is unique to the United Kingdom, and no longer in use today, it may require some explanation. Simply put, an ASBO was a civil order that could be given to any person age 10 or older—but in practice was primarily given to juveniles—who had been deemed to have acted in an antisocial manner. The ASBO was intended to deter future antisocial behavior by identifying (i.e., labeling) individuals judged to pose a risk to their community without having to resort to actual criminal sanctions. The process by which ASBOs were carried out resulted in an archetypal label—private citizens, such as neighbors in a housing development or local business owners, could file a complaint with the police that would result in another person being formally identified as someone likely to pose problems for the public. In essence, in cases where the police may not have been able to arrest or charge an individual, the community could undertake the process of public labeling of the individual as a risk to the community. The process required some evidence of community consensus to avoid application of an ASBO on the request of a sole complainant, which could have been subject to misuse. An ASBO could be used to deny services and entry to public spaces, and failure to comply with the ASBO could give rise to penalties that included criminal proceedings and sanctions (for more details on ASBOs, see Campbell, 2002).

Because the family fixed‐effects design is so robust, we can provide some of the most valid estimates of the impact of justice system contact on delinquency to date. If we find that spending a night in jail or prison, being issued an ASBO, or having an official crime record *decreases* later delinquency, then deterrence theory will be supported. If we find that contact with the justice system *increases* later delinquency, labeling theory will be supported.

## 1 BACKGROUND

### 1.1 Demonstrating crime does not pay

Deterrence theory is the foundation for many criminal justice policies and practices. Practices such as the process of being arrested (Sherman & Berk, 1984), incarcerated (Andenaes, 1968), and receiving an ASBO (Burney, 2005), as well as programs such as scared straight (Finckenauer, 1982), three‐strikes (Stolzenberg & D'Alessio, 1997), and Project HOPE (Cullen, Pratt, Turanovic, & Butler, 2018) are all built on the idea that use and threat of punishment promotes compliance with the law. Such ideas can be traced to the early philosophies of Beccaria (1963/1764) and Bentham (1948/1789) that came about during the eighteenth‐century Enlightenment. As such, deterrence theory is rooted in the assumption that humans are rational, hedonistic, and willing to express their free will. Humans weigh the benefits of offending against the costs of apprehension, recognizing that engaging in criminal acts may result in utility gain (e.g., pleasure or monetary rewards). But at the same time, the experience of coming into contact with the justice system may lead to a utility loss (e.g., physical or social pains and losses; Becker, 1968). Therefore, according to deterrence theory, when the justice system implements punishments that are swift, certain, and severe, it demonstrates to rational actors that crime does not pay because the expected loss of utility outweighs the expected utility gains (Matsueda, Kreager, & Huizinga, 2006; Moffitt, 1983; Tittle, 1975).

Teaching individuals that crime does not pay can be achieved in one of two ways. First, knowledge of the potential punishment one may receive by committing crime discourages society at large from engaging in criminal acts. This is known as “general deterrence,” and it suggests perceptions of punishment alone are enough to prevent the general public and would‐be offenders from becoming active criminals. The second method, known as “specific or individual deterrence,” indicates that the pains of punishments experienced by an active offender will discourage that particular individual from engaging in future criminal activity. Although both general and specific deterrence theories pose important hypotheses for scholars and policy makers to consider, we focused the current study on the latter—specific deterrence—by testing the following hypothesis:

 
*Deterrence hypothesis*: Spending time in jail or prison, receiving an ASBO, or having an official crime record will decrease later delinquency.

### 1.2 Criminogenic effects of the justice system

According to Tannenbaum (1938), state intervention and the application of labels leads to the “dramatization of evil” (pp. 19–20). “[T]he process of making the criminal, therefore,” Tannenbaum wrote, “is a process of tagging, defining, identifying, segregating, describing, emphasizing, making conscious and self‐conscious; it becomes a way of stimulating, suggesting, emphasizing, and evoking the very traits that are complained of” (p. 20). This leads to a process in which “the person becomes the thing he is described all as being” (p. 20). It is this proposition that sets the stage for labeling theory. In its simplest form, according to labeling theory, official responses to deviant behavior by the justice system will tend to increase the likelihood of future criminal involvement (e.g., Lemert, 1951; Tannenbaum, 1938). Ironically, then, the actions put forth by justice system officials to prevent crime will have the unintended consequence of increasing conduct problems (Cole, 1975; Cullen & Cullen, 1978; Schneider, 1975).

According to contemporary labeling theories, an increase in deviancy after justice system contact can arise from the experience of receiving a criminal label through two primary mechanisms (Paternoster & Iovanni, 1989). The first involves the structural impediments to conventional life that are the direct result of the label (see, e.g., Link, Cullen, Struening, Shrout, & Dohrenwend, 1989). For example, once convicted, offenders have a more difficult time finding steady employment as job applications are often rejected once the criminal record is revealed. Similarly, housing becomes difficult to find and the continuation of education is restricted as the ability to obtain student loans is limited as well as the ability to gain admittance to many universities (Stewart & Uggen, 2019). For juveniles, a reputation as having contact with the justice system might result in the young person being shunned by prosocial classmates or banned from the homes of prosocial friends by parents who worry the young person is a bad influence. Given that adolescence is a life stage when relationships with peers are exceptionally important, exclusion from prosocial groups might promote joining antisocial groups. These structural impediments restrict access to prosocial activities, prosocial relationships, and make a prosocial lifestyle more difficult, which in turn makes antisocial alternatives seem more viable, if not more attractive (e.g., see Bernburg & Krohn, 2003; Denver, Pickett, & Bushway, 2017; Kirk & Sampson, 2013; Widdowson, Siennick, & Hay, 2016; Wiley, Slocum, & Esbensen, 2013).

The second mechanism involves how a label can lead to a transformation of one's identity such that the new identity accepts a criminal lifestyle. Consistent with the thoughts of early labeling theorists (e.g., Lemert, 1951; Tannenbaum, 1938), the process of receiving a criminal label can lead to a self‐fulfilling prophecy (Merton, 1948) in which one internalizes his or her newly prescribed label. In this way, a criminal label leads to a fundamental shift in the character of an individual that leads to changes in his or her attitudes, personality, self‐concept, and sense of self‐worth (Becker, 1968; Matsueda, 1992). Contemporary labeling theorists have embraced the idea that justice system contact can change, or fix, an individual's narrative identity—the story of who the individual is, why it is so, and what his or her future holds. As Maruna (2001) explained, persistent offenders tend to embrace the story of a “condemnation script” where they feel hopelessness for their future and believe they have no other choice but to continue their offending behavior. Given such a lack of optimism, they will direct themselves to follow a trajectory of offending until they are capable of changing their narrative.

Through either (or both) pathways, according to labeling theory, when an individual receives a criminal or a delinquent label, the iatrogenic effects of that label will heighten his or her risk for becoming ensnared in a criminal career. Therefore, in opposition to deterrence theory, in labeling theory, justice system contact will heighten behavioral problems. In terms of the current study, then, labeling theory inspires the following research hypothesis:

 
*Labeling hypothesis*: Spending time in jail or prison, receiving an ASBO, or having an official crime record will increase later delinquency.

### 1.3 Deterrence, labeling, and individual differences

Adjudicating between the deterrence and labeling hypotheses would seem to be a straightforward task. Yet, the results from prior studies have been mixed. Although many study results seem to show favor for labeling theory (see, e.g., Chiricos, Barrick, Bales, & Bontrager, 2007; Krohn, Lopes, & Ward, 2014; Liberman, Kirk, & Kim, 2014; Petrosino, Turpin‐Petrosino, & Guckenberg, 2013), there is a body of evidence that demonstrates deterrent effects (see, e.g., Bhati & Piquero, 2007; Brennan & Mednick, 1994; Durlauf & Nagin, 2011; Fass & Pi, 2002). Other scholars, however, still have reported no effect of justice system contact on subsequent behavior or have reported mixed results (see, e.g., Morris & Piquero, 2013; Pratt, Cullen, Blevins, Daigle, & Madensen, 2006; Sherman, 1993). This reveals why Huizinga and Henry (2008, p. 245) concluded their systematic review by noting, “[T]he weight of the evidence suggests that arrest and sanctions either do not have much effect or increase subsequent delinquent behavior.”

Part of the reason for the mixed results is because contact with the justice system is not a stochastic, exogenous factor. To investigate the effects of justice system contact on future behavior, it is necessary to account for preexisting individual differences that may cause some individuals to have a higher risk of coming into contact with the justice system in the first place (e.g., see Murray, Farrington, & Eisner, 2009; Nagin & Paternoster, 1993; Piquero, Paternoster, Pogarsky, & Loughran, 2011; Pogarsky, 2002). Thus, the potential for confounding as a result of selection bias is important to recognize and control.

Smith and Paternoster (1990) conducted one of the first studies to acknowledge and correct for such biasing influences. These authors demonstrated that when examining delinquent outcomes between court‐referred and diverted youth, the results of a standard multiple regression analysis unveiled a labeling effect for the youth who had been court referred. Yet, once their models included controls for selection effects, no support for labeling was found.

Recently, propensity score matching (PSM) has emerged as a powerful method for identifying the effect of justice system contact on later behavior. PSM is capable of accounting for selection effects through its ability to match individuals on any observed characteristic (Loughran & Mulvey, 2008; Thoemmes & Kim, 2011). In this way, a researcher using PSM assumes matched cases mimic a counterfactual scenario, where each “treated” case (here, someone who has come into contact with the justice system) is matched with a similarly situated counterpart that has not received the “treatment.” The key to a successful PSM analysis rests on the researcher's ability to identify and measure all relevant confounding influences so that they can be used to make the matches.

The findings from studies in which PSM was employed tend to show support for the labeling hypothesis (but see McAra & McVie, 2007). The results of such analyses, for example, have demonstrated that individuals who experience police contact have an increased likelihood for future offending (Wiley & Esbensen, 2016; Wiley et al., 2013), including an increased likelihood for future violence (Ward, Krohn, & Gibson, 2014). Additionally, the findings from these PSM studies have shown that the experience of an arrest amplifies subsequent offending (Wiley & Esbensen, 2016; Wiley et al., 2013), especially for youth who are at the greatest risk for future antisocial behavior (Morris & Piquero, 2013).

Although PSM is a powerful technique, it has notable limitations. Most important is that matches between “treated” and “untreated” cases can only be made by drawing on characteristics that are observable and measured. As a result, there is the potential that unmeasured selection effects (i.e., unobserved confounding) have biased prior results.

With this in mind, we propose a slightly different strategy but one that can be situated in the counterfactual perspective that is the basis of PSM studies. The design we use—the family fixed‐effects model—can even be considered a “special case” of a PSM model. Pingault et al. (2018, p. 569) recently noted that the family fixed‐effects model can “approximate the counterfactual situation because a non‐exposed sibling or twin represents a natural match to their exposed co‐sibling or twin.” Siblings, and especially identical twins, can be thought of as a perfect “match” (in the language of PSM) for one another in terms of many environmental—as well as biological—factors (i.e., family confounds) that must be controlled to test our hypotheses.

Note that there are two types of twins: monozygotic (MZ) and dizygotic (DZ). MZ twins share 100 percent of their DNA while DZ twins share, on average, 50 percent. Both types of twins tend to share much of their rearing environment at least during their formative years (Barnes et al., 2014; Conley, Rauscher, Dawes, Magnussion, & Siegal, 2013). Recognizing these points, Kohler, Behrman, and Schnittker (2011, p. 91) noted that, “[T]wins have been extensively used to control for genetic and other background, unobserved, confounding factors. Social scientists have long used sibling comparisons for this purpose, reasoning that if brothers/sisters are similar with respect to family background and other characteristics, using differences between them…controls a great many relevant confounding factors.” Thus, twins and co‐twins can be matched, and whenever there is discordance on justice system contact, we can use that discordance to estimate the impact of justice system contact on later misbehavior. This is because the twin who has come into contact with the justice system can be considered the “treated” case and his or her co‐twin can be used to estimate the counterfactual.1We anticipate that the equal environment assumption (EEA) of twin studies will come to mind as a limitation of the family fixed‐effects model (see Burt & Simons, 2014). Although the EEA is specific to the estimation of heritability, which was not the focus of the present study, the essence of the EEA is important to keep in view. By design, our model rules out the influence of the shared environment. But to the extent that the EEA is violated, environmental influences will be nonshared between twins. Nonshared environments can be entered as covariates, which is why our fixed‐effects models include an extensive list of plausible nonshared environmental confounders.


Nedelec and Silver (2018) recently employed the family fixed‐effects model to estimate the impact of justice system contact on future criminal behavior among twins in the Add Health study. The authors reported null associations between various types of justice system contact and criminal offending in later adulthood. Thus, the findings from their study add to the mixed results that were noted earlier, albeit with a more powerful research design. Yet there are a few limitations to the Nedelec and Silver study that we will improve on in the present study. First, their measures of justice system contact included self‐reports of being stopped by the police and being arrested—events that occur at the beginning of the justice system funnel. In both deterrence and labeling theories, as one goes deeper into the system, the greater the effect on future behavior becomes. In the present study, we capture forms of justice system contact that occur at later stages in the process and are thus expected to be associated with more “pains” than those explored by Nedelec and Silver. We also include a mixture of self‐report and official records to address concerns over shared‐methods bias.

Second, Nedelec and Silver (2018) spoke to the long‐term effects of labeling because their measure of justice system contact was taken at least 6 years before their measure of criminal offending. Yet, at its core, labeling theory (and deterrence theory) is primarily concerned with the temporally proximate effect of justice system contact that may create long‐term consequences. In our study, we have a much narrower window of time between the independent and dependent variables, which allows us to observe better the proximate effects of justice system contact.

Third, because Nedelec and Silver (2018) analyzed the adulthood phases of the Add Health data, offending rates were low, which raises potential concerns about statistical power. We focus our analysis on a broad spectrum of self‐reported delinquency measured during adolescence as the outcome. Thus, we avoid the problem of low base rates and, in so doing, perform an analysis that is more closely aligned with the deterrence and labeling hypotheses that were outlined earlier. Fourth, we draw on a data set that has a larger number of twin pairs (about twice as large) than the Add Health sample, thus, improving statistical power and the precision in our estimates.

## 2 DATA AND METHOD

### 2.1 Sample

We analyze data from the Environmental Risk (E‐Risk) Longitudinal Twin Study, which is a longitudinal and nationally representative study in which the development of a birth cohort of 2,232 same‐sex British twins are tracked who were sampled from a birth registry of twins born in England and Wales from 1994 through 1995 (Trouton, Spinath, & Plomin, 2002). Full details of the E‐Risk sample are described elsewhere (see Moffitt & E‐Risk Study Team, 2002). To summarize, the sample was constructed in 1999 to 2000 when 1,116 families (93 percent of those eligible) with same‐sex 5‐year‐old twins participated in home‐visit assessments. The full sample was evenly distributed across sex (49 percent male) and comprised 56 percent monozygotic (MZ; identical) and 44 percent dizygotic (DZ; fraternal) twin pairs. Families were recruited to represent the U.K. population of families with newborns in the 1990s, based on residential location throughout England and Wales and mother's age. Older mothers having twins via assisted reproduction were undersampled to avoid an excess of well‐educated older mothers, whereas teenage mothers with twins were oversampled to ensure sufficient numbers of children growing up in high‐risk environments, and replace teen‐mother families lost to the original register as a result of nonresponse. These strategies ensured that the study sample was representative of the full range of socioeconomic conditions in the United Kingdom, as reflected in the families’ distribution on a neighborhood socioeconomic index (A Classification of Residential Neighbourhoods [ACORN], developed by CACI, Inc., for commercial use in Great Britain; Odgers et al., 2012).2ACORN uses census and other survey‐based geodemographic discriminators to classify enumeration districts of approximately 150 households into socioeconomic groups. Such groups range from “wealthy achievers” with high incomes, large single‐family houses, and access to many amenities, to “hard‐pressed” neighborhoods dominated by government‐subsidized housing estates, low incomes, high unemployment, and single parents. ACORN classifications were geocoded to match the location of each E‐Risk study family's home (Odgers, Caspi, Bates, Sampson, & Moffitt, 2012). E‐Risk families’ ACORN distribution closely matches that of households nationwide: 25.6 percent of E‐Risk families live in “wealthy achiever” neighborhoods compared with 25.3 percent nationwide, 5.3 versus 11.6 percent live in “urban prosperity” neighborhoods, 29.6 versus 26.9 percent live in “comfortably off” neighborhoods, 13.4 versus 13.9 percent live in “moderate means” neighborhoods, and 26.1 versus 20.7 percent live in “hard‐pressed” neighborhoods. It should be noted that the underrepresentation of “urban prosperity” in E‐Risk is because such households are significantly more likely to be childless. See figure S1 in the online supporting information for further details about the representativeness of the E‐Risk participants compared with the United Kingdom as a whole.3Additional supporting information can be found in the full text tab for this article in the Wiley Online Library at http://onlinelibrary.wiley.com/doi/10.1111/crim.2020.58.issue-2/issuetoc.


Follow‐up home visits were conducted when the participants were aged 7 (98 percent participation), 10 (96 percent), 12 (96 percent), and 18 (93 percent). The home visits at ages 5, 7, 10, and 12 years included assessments with the participant as well as their mother (or primary caretaker). Each twin participant was assessed by a different interviewer. With parent's permission, questionnaires were posted to the children's teachers. At age 18 years, 2,066 participants were assessed. These home visits included interviews only with the participants. There were no differences between those who did and did not take part at age 18 in terms of socioeconomic status (SES) assessed when the cohort was initially defined (χ^2^ = .86, *p* = .65), age 5 IQ scores (*t* = .98, *p* = .33), or age 5 externalizing behavioral (*t* = .40, *p* = .69) or internalizing emotional problems (*t* = .41, *p* = .68). Parents gave informed consent, and twins gave assent between ages 5 and 12. Twins gave informed consent at age 18 years. Ethical approval for each phase of the study was granted by the Joint South London and Maudsley and the Institute of Psychiatry NHS Ethics Committee. After removing cases with missing values on our focal variables, our final analytical sample was *n *= 1,903 individuals (comprising 901 twin pairs).4As explained in the *crime record* description, we dropped *n *= 69 cases as a result of ambiguous temporal ordering. No variable had more than 5 percent missing: *delinquency at age 12* (*n* = 63), *externalizing problems at age 12* (*n* = 52), *low self‐control* (*n* = 0), *cognitive ability at age 12* (*n* = 55), and *educational achievement* (*n* = 5). Also, our data were slightly unbalanced (in some families, one twin was missing data but the co‐twin was not). Unbalanced data do not bias regression coefficients in fixed‐effects models (Allison, 2009). Robustness checks confirmed that coefficient estimates were not impacted when the data were restricted to fully balanced pairs.


### 2.2 Measures

#### 2.2.1 Dependent variable

##### Delinquency at age 18

The dependent variable for this study was a variety index of delinquent behaviors taken from the age‐18 interview. Self‐reported involvement in delinquency was assessed through a computer questionnaire. The monitor was positioned so that the twin saw the questions in private and twins wore headphones to hear each question aloud to assist poor readers. Twins were instructed to report on their behavior over the past 12 months. The specific delinquent offenses included fighting (reported by 10 percent of participants), bullying (13 percent), being cruel to others (9 percent), being cruel to animals (2 percent), using weapons (2 percent), vandalism (19 percent), lying (42 percent), robbery (1 percent), shoplifting (26 percent), breaking into homes or cars to steal (6 percent), running away (10 percent), and truancy (35 percent). Responses were coded *no* = 0 and *yes* = 1, such that summing across the 13 items created a variety index. The resulting index had a mean of 2.05, had a standard deviation of 2.25, and ranged between 0 and 11.

It should be noted that the findings from several studies have demonstrated the reliability and validity of self‐reported criminal behavior and justice system involvement in samples from prospective longitudinal studies that are similar in design to the E‐Risk (Auty, Farrington, & Coid, 2015; Henry, Moffitt, Caspi, Langley, & Silva, 1994; Krohn, Lizotte, Phillips, Thornberry, & Bell, 2013; Pollock, Mendard, Elliott, & Huizinga, 2015). Such conclusions drawn from these studies should provide confidence to the results gleaned in the current study. Some readers, however, may be concerned that our measure of delinquency includes behaviors or acts that are not technically illegal. To account for this, we estimated supplemental models in which the outcome variable was constructed using only the illegal delinquent behaviors (see tables S1–S3 and S7–S9 in the online supporting information). The results from these analyses were substantively similar to those presented in the main text using the full measure of delinquency.

It is important to note that observations varied both *between* families of twins (standard deviation = 2.03) and *within* twin pairs (standard deviation = 1.07). There was greater variation at the *between*‐family level, which indicates important family effects should be controlled. These are precisely the influences that are controlled by design in the family fixed‐effects model. Thus, our analysis is focused exclusively on explaining sources of variation that is observed within twin pairs while adjusting for all between‐family influences. Summary statistics for all measures are presented in table 1.

**Table 1:** Descriptive statistics

	Full Sample	MZ Twins	DZ Twins
	Mean [Proportion]	Mean [Proportion]	Mean [Proportion]
Variables	(SD)	(SD)	(SD)
Dependent Variable			
Delinquency at age 18	2.050	1.988	2.131
	(2.247)	(2.292)	(2.185)
Key Independent Variables			
Jail/prison = 1	[.053]	[.051]	[.056]
	—	—	—
ASBO = 1	[.014]	[.015]	[.013]
	—	—	—
Crime record = 1	[.071]	[.072]	[.071]
	—	—	—
Covariates			
Delinquency at age 12	2.425	2.431	2.418
	(2.934)	(2.984)	(2.870)
Ext. problems at age 12	15.006	15.029	14.976
	(14.123)	(14.002)	(14.284)
Low self‐control	−.052	−.018	−.095
	(.984)	(.997)	(.965)
Cognitive ability at age 12	100.000	99.390	100.781
	(15.000)	(14.575)	(15.500)
Educational achievement	2.274	2.303	2.237
	(.848)	(.833)	(.866)
*N*	1,903	1,068	835

*Abbreviations*: ASBO = anti‐social behaviour order; DZ = dizygotic; Ext. = externalizing; MZ = monozygotic; SD = standard deviation (omitted for binary variables).

#### 2.2.2 Key independent variables

##### Jail/prison

During the age‐18 interview, each participant was asked the following: “Have you ever had to spend a night in police custody, jail, or prison?” The reporting period covered age 10 up to age 18. Responses were coded such that *no *= 0 and *yes* = 1. Approximately 5 percent (*n* = 101) of participants reported having spent time in jail or prison. This value is consistent with estimates of the prevalence of incarceration among young adults from the United States (Barnes, 2014). Importantly, we observed *within* twin pair discordance: Of families in which at least one twin had spent a night in jail/prison, 70 percent (48 of 69 families) were discordant for this experience.5It should be noted that the total prevalence of those who reported having spent time in jail or prison includes all cases, regardless of whether the co‐twin also had available data. Only balanced twin pairs are used to report discordance (this is also true for the *ASBO* and *crime record* discussions. Furthermore, when restricted to MZ twin pairs, 68 percent (26 of 38 families) of families in which at least one twin had spent a night in jail/prison were discordant.

##### Anti‐social behaviour order (ASBO)

During the age‐18 interview, each participant was asked the following: “Have you ever been issued an ASBO (Anti‐social Behaviour Order)?” (*no *= 0, *yes* = 1). ASBOs were introduced in the United Kingdom in 1999 when the E‐Risk sample had reached 4–5 years old. According to the Home Office, “ASBOs are civil orders that exist to protect the public from behaviour that causes or is likely to cause harassment, alarm or distress. An order contains conditions prohibiting the offender from specific anti‐social acts or entering defined areas and is effective for a minimum of two years. The orders are not criminal penalties and are not intended to punish the offender.” (Home Office, 2003, p. 9). Approximately 1.4 percent (*n *= 27) of twins reported being issued an ASBO, and there was within‐twin pair discordance: 65 percent (11 of 17 families) of families in which at least one twin had been issued an ASBO were discordant for this outcome (60 percent [6 of 10 families] of MZ twin pairs were discordant). The reporting period was “ever” from age 10 up to age 18.

##### Crime record

Official records of participants’ cautions and convictions were obtained through United Kingdom Police National Computer (PNC) record searches conducted in cooperation with the United Kingdom Ministry of Justice. Records include the complete histories of cautions and convictions in the United Kingdom beginning when participants were age 10 years (the age of criminal responsibility). Cautions can be thought of as warnings given by the police for minor crimes. To receive a caution, an individual has to admit to the offense and accept the warning. Although a caution is not a criminal conviction, failure to express guilt and agree to the terms of the caution can lead to a criminal conviction. Additionally, cautions can be used as evidence for previous criminal behavior if an individual happens to go to trial for a different crime.

Cautions and convictions for the E‐Risk data are date stamped and currently complete through age 19 years. Therefore, to establish the correct temporal ordering with the dependent variable, we coded these data into a binary variable that reflected whether participants had been cautioned or convicted (= 1) or not (= 0) before age 17 years.6In the current study, we combined cautions and convictions into a single item. Participants, however, experienced cautions only and convictions in nearly an equal ratio. In all, approximately 7 percent (*n* = 136) of E‐Risk participants had been cautioned or convicted before age 17. For families in which at least one twin was cautioned or convicted before age 17, 68 percent (61 of 90 families) were discordant (59 percent [*n *= 29 of 49 families] of MZ twin pairs were discordant).

In an effort to avoid biasing the coefficient of relationship between the *crime record* variable and *delinquency*, we removed from the sample any participant who had received a caution or conviction after their seventeenth birthday but not before. This restriction was necessary because, for those cases, the temporal ordering of the independent and dependent variables was ambiguous. For example, a participant celebrating his or her eighteenth birthday could have engaged in delinquency and been cautioned or convicted for those acts. The delinquent behavior would have registered in our *delinquency *outcome, but the participant would have been coded 0 on the *crime record *variable. Retaining cases like this would bias our coefficient estimates. Also, removing these cases had the added benefit of reducing ambiguity in the temporal ordering of the* jail/prison *variable and the *ASBO* variable by removing cases in which these events possibly overlapped with a caution or conviction during the delinquency reporting period. In all, *n*  = 69 participants were removed from the analytical sample as a result of this restriction (all descriptive statistics reported above and in table 1 were computed after removing these ambiguous cases).

#### 2.2.3 Covariates

Although the family fixed‐effects design rules out many sources of confounding, it is necessary to control for confounding influences that are not shared by twins, such as cognitive and behavioral differences (Beaver, 2008; Harris, 1995; Knopik, Neiderhiser, DeFries, & Plomin, 2017). These nonshared factors can be thought of as influences that are not equal across twins, including environments that violate the equal environments assumption (see footnote 1). To address this point, we included several covariates in our multivariate analyses. Also, it should be noted the E‐Risk comprises exclusively of same‐sex pairs, so sex is controlled by design in the family fixed‐effects models. For a full description of the measurement details of the covariates, see appendix A at the end of this article.

Self‐reported *delinquency at age 12* was included to capture involvement in delinquency from the previous assessment period. A composite scale of mother‐ and teacher‐reported *externalizing problems at age 12* was also included. This measure combines information from the parent‐reported Child Behavior Checklist (Achenbach, 1991a) and information from the Teacher's Report Form (Achenbach, 1991b). Notably, parents (most frequently mothers) and teachers reported on a range of behaviors, including each individual twin's exposure to antisocial peers. A multi‐occasion/multi‐informant factor tapping into *low self‐control* during the twin's first decade of life was included to capture individual differences as a result of levels of self‐control. *Cognitive ability at age 12* was measured via the Wechsler Intelligence Scale for Children (2003). At age 18, participants were classified into four groups according to the highest educational qualification they attained based on their performance on the General Certificate of Secondary Education. These standardized examinations are taken by students at the end of compulsory education at 16 years of age. We included the group each participant sat in as the *educational achievement* variable in our analysis. Finally, the analysis includes a control for a parent‐reported indicator of which twin was born first within the pair (*twin identifier*).

### 2.3 Analysis plan

The analysis proceeded in three interrelated steps, with each step progressively accounting for more potential sources of confounding. All associations presented in the tables in this section were estimated by linear regression. Because the outcome variable (*delinquency at age 18*) showed signs of positive skew, we conducted robustness checks using Poisson regression. We chose not to present them as our feature results because the Poisson model, when estimated in a family fixed‐effects framework, drops any cases in which twin 1 and twin 2 both scored 0 on the outcome (Rabe‐Hesketh & Skrondal, 2012). This resulted in *n *= 308 cases being omitted from the Poisson analysis. Rather than lose those cases, we opted to estimate all relationships by linear regression. Importantly, though, our substantive conclusions remain the same when the relationships are estimated by Poisson regression (see tables S4–S6 in the online supporting information).

The first step estimated the association between contact with the justice system and delinquency at age 18. Separate models were analyzed for our three measures of contact with the justice system: ever spending a night in jail/prison, ever being issued an ASBO, and having an official crime record before age 17. In these models, a statistically significant negative association indicates that contact with the justice system predicts *lower* levels of delinquency at age 18 (i.e., a deterrent effect). A positive association indicates that justice system contact predicts *higher* levels of delinquency at age 18 (i.e., a labeling effect). Multivariate models were statistically adjusted for the covariates listed earlier. These models were not, however, designed to account for family‐level effects. In this respect, we consider the estimates from these models similar to those that have been produced by prior research.

The second and third steps of the analysis had a family fixed‐effects design. This model can be expressed algebraically as follows (see Kohler et al., 2011; for a general discussion of the standard fixed effects regression model, see Allison, 2009; Wooldridge, 2015):

$$y_{1j}-y_{2j}=\left(\mu_{1}-\mu_{2}\right)+b_{1}\left(x_{1j}-x_{2j}\right)+b_{2}\left(c_{1j}-c_{2j}\right)+\left(e_{1j}-e_{2j}\right)$$

where *y*_1_ *_j_* – *y*_2_ *_j_* captures the within‐twin pair difference in delinquency between twin 1 and twin 2 in family *j*, *μ*_1_ – *μ*_2_ represents the mean within‐twin pair difference observed in the sample, *b*_1_ reflects the effect of within‐twin pair differences in the key independent variable on the outcome, *b*_2_ represents the collective influence of the covariates, and *e*_1_ *_j_* – *e*_2_ *_j_* estimates the impact of unmeasured within‐twin pair differences on the outcome.

For the second step of our analysis, we estimated the effect of contact with the justice system on delinquency using the family fixed‐effects model. We did so using the full sample, which includes both MZ and DZ twin pairs. Use of this model allowed for us to observe the effect of contact with the justice system on delinquency at age 18 while statistically controlling for family effects (meaning anything that is shared by twins that works to make them more similar to one another) and, in the multivariate model, the observed environmental factors captured by the covariates. Yet the inclusion of DZ twins in the sample does not allow for us to adjust for genetic endowments in full because DZ twins only share, on average, 50 percent of their DNA.

In the third step of the analysis, we also relied on the family fixed‐effects model, but this time the sample was restricted to MZ twin pairs. This analysis produced the most conservative coefficient estimates because it ruled out all family‐level effects, including genetic endowments. We will draw on the pattern of findings from all three steps to reach substantive conclusions concerning the degree of support we find for deterrence or labeling theory.

## 3 RESULTS

### 3.1 Does spending a night in jail or prison increase or decrease delinquency?

Table 2 shows the results of the statistical models using the *jail/prison* variable as the key predictor of *delinquency at age 18*. The table includes six columns, with each column presenting estimates from a unique model. The first column in the table, labeled “No Fixed Effects”, presents the results of a bivariate ordinary least‐squares (OLS) regression model. As the label indicates, this model does not include family fixed effects. As can be seen, consistent with labeling theory's hypothesis, spending a night in jail/prison was associated with higher levels of delinquency at age 18 and the effect was statistically significant (*p* < .001). The effect size estimate was *b* = 3.648, indicating that youth who reported spending a night in jail/prison self‐reported more than 3.5 more acts of delinquency at age 18, on average, compared with youth who had not spent a night in jail/prison.

**Table 2:** Regression of delinquency at age 18 on spent night in jail/prison and covariates

	No Fixed Effects		Fixed Effects		Fixed Effects	
	(MZ & DZ)		(MZ & DZ)		(MZ Only)	
	*b*	*b*	*b*	*b*	*b*	*b*
Variables	[95% CI]	[95% CI]	[95% CI]	[95% CI]	[95% CI]	[95% CI]
Key Independent Variable						
Jail/prison = 1	3.648***	2.396***	1.958***	1.627***	1.808***	1.745***
	[3.060,4.236]	[1.789,3.003]	[1.346,2.571]	[1.026,2.229]	[.992,2.623]	[.948,2.542]
Covariates						
Delinquency, age 12	—	.247***	—	.147***	—	.157***
		[.198,.296]		[.100,.195]		[.092,.221]
Ext. problems, age 12	—	.018***	—	.018**	—	.009
		[.008,.029]		[.005,.030]		[–.009,.027]
Low self‐control	—	.204**	—	.135	—	.436**
		[.081,.327]		[–.046,.316]		[.124,.748]
Cognitive ability, age 12	—	.015***	—	.006	—	.005
		[.008,.022]		[–.006,.017]		[–.012,.023]
Educational achievement	—	–.090	—	–.104	—	–.072
		[–.225,.045]		[–.297,.090]		[–.361,.216]
Twin identifier	—	–.138	—	–.098	—	–.100
		[–.280,.004]		[–.235,.040]		[–.278,.079]
Family fixed effects included?	No		Yes		Yes	
*N*	1,903		1,903		1,068	

*Abbreviations*: CI = confidence interval; DZ = dizygotic twin pairs; Ext. = externalizing; MZ = monozygotic twin pairs.

*p < .05; **p < .01; ***p < .001 (two‐tailed).

The second OLS model under the “No Fixed Effects” heading was adjusted for the influence of the covariates. Notably, among the covariates is a measure of delinquency taken from age 12. Thus, the multivariate analysis estimates can be interpreted as the impact of spending a night in jail/prison on *changes* in delinquency between age 12 and 18. After including the covariates, we see the positive effect remains, albeit reduced in size. Specifically, spending a night in jail or prison is associated with a 2.396 (*p* < .001) point increase, on average, in delinquency from age 12 to age 18.

The two columns in the middle of table 2 present estimates gleaned from the family fixed‐effects models where both MZ and DZ twin pairs were included. These coefficient estimates can be interpreted as the impact of within‐twin pair differences in ever spending a night in jail/prison on twin‐pair differences in delinquency at age 18. The results of the bivariate model presented in the third column reveal that, after accounting for family fixed effects, twins who reported spending a night in jail or prison self‐reported *b* = 1.958 (*p* < .001) more acts of delinquency, on average, compared with their co‐twin who did not spend a night in jail or prison. In other words, after accounting for family fixed effects, we maintain support for the labeling hypothesis. The statistical and substantive conclusions do not change when the covariates are added to the model (*b* = 1.627, *p* < .001).

Based on these results, we were concerned that the observed effects may represent a general deterrent/spillover effect (see, generally, Bhuller, Dahl, Løken, & Mogstad, 2018), where the twin who experienced justice system contact may not change his or her level of delinquency at age 18, but the co‐twin reduced his or her involvement in delinquency. This would substantively reveal a general deterrent effect but would statistically appear as a labeling effect. We, therefore, performed a supplemental analysis by computing the residuals from a regression model where delinquency at age 18 was the dependent variable and delinquency at age 12 was the predictor. The pattern of residuals we observed indicated that, on average, twins who had spent a night in jail/prison had large positive residuals (indicating an increase in delinquency), whereas their co‐twins who did not have contact tended to have residuals that were statistically indistinguishable from zero. This finding bolsters our conclusion that the positive coefficients lend support for a labeling effect instead of a scenario where there was a general deterrent/spillover effect from one twin to the other.

Returning to table 2, we see the estimate was not substantively affected when the analysis was restricted to MZ twins in the “Fixed Effects (MZ Only)” models, which appear in the last two columns of table 2. After accounting for all sources of family effects and the covariates, MZ twins who had spent a night in jail or prison, on average, increased their involvement in delinquency by 1.745 points (*p* < .001) from age 12 to age 18 compared with their co‐twin who had not spent a night in jail or prison. Taken together, the positive associations between ever spending a night in jail/prison and delinquency at age 18 in all of the models provides robust evidence in favor of the labeling hypothesis.

### 3.2 Does being issued an anti‐social behaviour order (ASBO) increase or decrease delinquency?

Table 3 shows the results of the statistical models using the *ASBO* variable as the key predictor of *delinquency at age 18*. Again, consistent with the labeling hypothesis, being issued an ASBO was associated with an increase in delinquency at age 18 in the OLS regression models (the “No Fixed Effects” columns). Estimates from the multivariate model indicated that, on average, individuals who had been issued an ASBO showed greater increases from age 12 to age 18 than did those who had not been issued an ASBO (*b* = 2.751, *p* < .001).

**Table 3:** Regression of delinquency at age 18 on being issued an ASBO and covariat**es**

	No Fixed Effects		Fixed Effects		Fixed Effects	
	(MZ & DZ)		(MZ & DZ)		(MZ Only)	
	*b*	*b*	*b*	*b*	*b*	*b*
Variables	[95% CI]	[95% CI]	[95% CI]	[95% CI]	[95% CI]	[95% CI]
Key Independent Variable						
ASBO = 1	4.232***	2.751***	3.091***	2.990***	3.667***	3.978***
	[3.180,5.284]	[1.646,3.856]	[1.799,4.383]	[1.728,4.253]	[1.967,5.366]	[2.332,5.625]
Covariates						
Delinquency, age 12	—	.263***	—	.162***	—	.161***
		[.214,.312]		[.114,.210]		[.097,.225]
Ext. problems, age 12	—	.022***	—	.017**	—	.010
		[.012,.033]		[.005,.029]		[−.009,.028]
Low self‐control	—	.198**	—	.110	—	.467**
		[.073,.322]		[−.072,.292]		[.156,.778]
Cognitive ability, age 12	—	.016***	—	.007	—	.004
		[.009,.023]		[−.004,.019]		[−.013,.021]
Educational achievement	—	−.187*	—	−.185	—	−.163
		[−329,−.044]		[−.378,.007]		[−.449,.123]
Twin identifier	—	−.151*	—	−.112	—	−.120
		[−.293,−.009]		[−.250,.025]		[−.299,.058]
Family fixed effects included?	No		Yes		Yes	
*N*	1,903		1,903		1,068	

*Abbreviations*: ASBO = anti‐social behaviour order; CI = confidence interval; DZ = dizygotic twin pairs; Ext. = externalizing; MZ = monozygotic twin pairs.

*p < .05; **p < .01; ***p < .001 (two‐tailed).

The family fixed‐effects estimates corroborated the findings from the OLS models by revealing that after adjusting for family effects, twin‐pair differences in being issued an ASBO were statistically significantly associated with twin‐pair differences in delinquency at age 18. Twins who had been issued an ASBO, on average, increased in delinquency from age 12 to age 18 by 2.990 points (*p* < .001). The findings from supplemental analysis again revealed that the positive coefficient observed here was a result of an increase in delinquency on the part of the twins who had received an ASBO and not because of a decrease in delinquency on the part of the twins who did not receive an ASBO. Specifically, on average, the residuals generated from a regression model predicting delinquency at age 18 with delinquency at age 12 were large and positive for twins who had been issued an ASBO and were statistically indistinguishable from zero for co‐twins who had not been issued an ASBO. Furthermore, substantive conclusions were robust to further controls for genetic influences in the “MZ Only” fixed‐effects model (last two columns in table 3), providing consistent support for the labeling hypothesis.

### 3.3 Does having an official crime record increase or decrease delinquency?

Table 4 shows the results of the statistical models using the official *crime record* variable as the key predictor of *delinquency at age 18*. We consider the results from this section to be the most conservative because they rely on the official Ministry of Justice records, which allows for us to rule out shared‐methods bias and to ensure proper temporal ordering of all variables.

**Table 4:** Regression of delinquency at age 18 on having an official crime record before age 17 and covariates

	No Fixed Effects		Fixed Effects		Fixed Effects	
	(MZ & DZ)		(MZ & DZ)		(MZ Only)	
	*b*	*b*	*b*	*b*	*b*	*b*
Variables	[95% CI]	[95% CI]	[95% CI]	[95% CI]	[95% CI]	[95% CI]
Key Independent Variable						
Crime record = 1	2.329***	1.065***	.984***	.681*	.828*	.694
	[1.778,2.881]	[.498,1.633]	[.432,1.535]	[.144,1.217]	[.044,1.611]	[−.082,1.469]
Covariates						
Delinquency, age 12	—	.258***	—	.146***	—	.151***
		[.209,.308]		[.098,.195]		[.086,.216]
Ext. problems, age 12	—	.020***	—	.019**	—	.010
		[.010,.031]		[.007,.032]		[−.009,.029]
Low self‐control	—	.209**	—	.142	—	.434**
		[.084,.334]		[−.041,.325]		[.117,.751]
Cognitive ability, age 12	—	.016***	—	.007	—	.007
		[.009,.023]		[−.004,.019]		[−.011,.025]
Educational achievement	—	−.168*	—	−.175	—	−.187
		[−.312,–.023]		[−.369,.019]		[−.481,.107]
Twin identifier	—	−.134	—	−.094	—	−.081
		[–.277,.010]		[–.233,.045]		[–.263,.101]
Family fixed effects included?	No		Yes		Yes	
*N*	1,903		1,903		1,068	

*Abbreviations*: CI = confidence interval; DZ = dizygotic twin pairs; Ext. = externalizing; MZ = monozygotic twin pairs.

*p < .05; **p < .01; ***p < .001 (two‐tailed).

Consistent with the labeling hypothesis, having a crime record was associated with an increase in delinquency at age 18 in the OLS regression models (the “No Fixed Effects” columns). Estimates from the multivariate model indicated that, on average, individuals who had a crime record showed greater increases in delinquency from age 12 to age 18 than did those who did not have a record (*b* = 1.065, *p* < .001).

The family fixed‐effects estimates substantively fall in line with those from the OLS models by demonstrating that after adjusting for family effects, twin‐pair differences in having a crime record were significantly associated with twin‐pair differences in delinquency at age 18 in the full sample. Twins who had a crime record, on average, increased in delinquency from age 12 to age 18 by .681 points (*p* < .05). Again, the findings from supplemental analysis revealed the positive coefficient observed here was a result of an increase in delinquency on the part of the twins who had a crime record and not because of a decrease in delinquency on part of the twins who did not. Specifically, on average, the computed residuals for twins who had a crime record were positive, whereas the residuals for co‐twins without a crime record were not statistically distinguishable from zero.

It should be noted, though, that the size of the coefficient is reduced as we move to the fixed‐effects models more so for this variable than for the *jail/prison* and *ASBO* variables. This pattern of results indicates some selection effects for the *crime record* variable are ruled out by controlling for family‐level influences. Although we cannot know for sure, some family‐level effects (e.g., neighborhood influences or extralegal influences) may have been shared by twins that make it more/less likely they will have an official record. This finding could be the explanation for the coefficients showing a more substantial decrease from the OLS model to the family fixed‐effects models here than when the other two justice contact variables were examined. Nonetheless, the coefficient estimates gleaned from the family fixed‐effects models are substantively significant and stable from the full sample model to the MZ‐only model, although it falls just outside of the conventional threshold for statistical significance (*b *= .694, *p* = .079) in the multivariate MZ‐only model (see the last columns in table 4). It is also important to note that we observed a positive and statistically significant coefficient when the dependent variable was restricted to illegal delinquency (results presented in tables S1–S3 and S7–S9 of the online supporting information). Thus, when taken together, the evidence from these analyses show support for the labeling hypothesis.

## 4 DISCUSSION AND CONCLUSION

We sought to conduct a rigorous test between deterrence and labeling hypotheses. Drawing on data from a nationally representative and longitudinal birth cohort of British adolescent twins, we found that contact with the justice system—through spending a night in jail/prison, being issued an ASBO, or having an official crime record—promotes misbehavior, which supports the labeling hypothesis. With this in mind, we highlight four contributions from this study that warrant consideration. We then discuss some of the broader implications our findings might have for the justice system.

### 4.1 Contributions

First, we followed the call of previous research (see, e.g., Farrington, 2003; Murray et al., 2009; Piquero et al., 2011; Pogarsky, 2002) and employed one of the most rigorous nonexperimental methodological designs capable of accounting for a wide range of selection effects and confounding influences. Using the family fixed‐effects model (Kohler et al., 2011), we leveraged nationally representative twin data to take advantage of the natural experiment that twins provide by the fact that they share their family environment and their genetic endowments. Such family effects work to make twins similar to one another. By focusing on within‐twin pair differences, then, we were able to rule out the effects of these family environments and genetic influences, providing us the opportunity to glean some of the most precise estimates for the impact of justice system contact on future behavior. In doing so, we have demonstrated that twin samples and methods have utility for criminological theory testing that reaches beyond the typical strategy of estimating heritability (see Moffitt & Beckley, 2015).

A second feature of this study is that we drew on three separate measures—two that were self‐reported and one obtained from official Ministry of Justice records—of justice system contact. The pattern of findings was substantively consistent across these specifications, providing robust support for the labeling hypothesis. The findings across such forms of contact demonstrate that even sanctions that do not penetrate far into the justice system are potentially criminogenic, an outcome that has important implications for policy. Of interest to labeling theorists, the effect of ASBO was found to be a substantively strong predictor of later misbehavior. This is important because, in our opinion, the ASBO represented an archetypal label—recall that it was not intended to be punitive; rather, it was intended to be preventative by identifying those who were at risk of bad behavior. It was also intended to be a public label, and that is exactly the effect it seemed to have had. We believe the findings from the ASBO analysis are particularly revealing given this context even though ASBOs are no longer in use.

Third, we analyzed as an outcome broad‐spectrum delinquency rather than an official outcome (e.g., rearrest or reconviction) that is more commonly assessed in the deterrence and labeling literatures. The results for justice system outcomes like rearrest may be biased because individuals who experience such contact are often at an increased risk for future contact with the justice system simply because they are known by its actors, such as arresting police officers. An outcome variable such as delinquency, therefore, allowed for us to observe change in behavior that is not biased by the actions of justice system actors. Furthermore, by relying on self‐reported delinquency, we can capture delinquent and illegal acts done by the participants that may not be known to the justice system, which would not be captured if we were to rely on official records. For these reasons, we believe the focus on self‐reported delinquency represents an important contribution to the labeling literature.

Fourth, we relied on a sample of individuals who are within the primary age range for engaging in antisocial behavior (i.e., 18‐year‐olds). This is important as it captures the impact of justice system contact for those who are peaking in their criminal careers. The impact of such contact for this population is notable as the increase in problem behavior may lead to a downward spiral of cumulative continuity for certain youth (e.g., Caspi, Bem, & Elder, 1989; Moffitt, 1993; Nagin & Paternoster, 1991; Sampson & Laub, 1992). Yet it should be noted that, at this time, we cannot observe how the increases in scores for delinquency will go on to affect participants’ criminal trajectories. Follow‐up analyses of this cohort with future phases of data collection will be better suited to answer that question.

### 4.2 Limitations and future directions

Although this study has many strengths, it should be kept in mind that participants were asked whether they had ever been in jail/prison or had ever been issued an ASBO during the age‐18 interviews. Participants were not asked how old they were when these events occurred (E‐Risk did not ask about timing because of concerns of telescoping when individuals are asked to report on open‐ended events [Henry et al., 1994; Moffitt, Caspi, Rutter, & Silva, 2001]). Thus, in theory, it is possible that some participants spent a night in jail or prison or were issued an ASBO during the same past‐year period that they self‐reported on their delinquency. In such individuals, the high rate of delinquency reported at age 18 might have precipitated the jail or prison night or the ASBO, complicating interpretations of the direction of influence between the two. Yet, we think this is unlikely to characterize many participants, especially for ASBOs, given that we know from other sources (newspapers, Police National Computer) that the earliest ASBOs in the United Kingdom, and in the E‐Risk cohort, were given at age 10. Recall, however, that we were able to rule out this possibility for our analyses using the official record variable (cautions/convictions) and the substantive pattern of findings was unaffected. The estimated effect size, however, was smaller for the official record variable compared with the estimated effect sizes for jail/prison and ASBO.

An additional limitation is that this study was focused on one cohort of twins from the United Kingdom. Findings should be replicated using twins (or siblings) from other countries and potentially from other birth cohorts to gain a broader perspective on how generalizable the results are.

With the results and the associated limitations of our study in mind, we believe there are at least three areas on which future studies can build. First, we were able to speak to the average effect of justice system contact on subsequent delinquent behavior. There is now a need for insight into whether there is heterogeneity in the impact of justice system contact experiences. In particular, scholars should attempt to disentangle the differential effects potentially produced via dosage differences (e.g., length of time spent incarcerated and number of times incarcerated/convicted), legal differences (e.g., type of crime and age at offense), and differences in the offender's perception of the punishment (e.g., procedural justice). There is also a need to test offense‐specific hypotheses in the deterrence/labeling debate. For instance, what effect does justice system contact have on the behaviors that caused the initial contact/sanctioning? We were unable to test offense‐specificity, and other researchers in the deterrence versus labeling literature have also been unable to do so. This open area of inquiry, therefore, should be explored.

Second, even though we believe the family fixed‐effects model is one of the most robust methodologies for investigating the influence of contact with the justice system, we do believe the model could be improved by embedding the family fixed‐effects model within the longitudinal fixed‐effects model. Such an analysis would allow researchers to control, by design, the environments that are shared within‐twin pairs as well as the time‐stable nonshared environmental influences. In essence, this would generate a three‐level model in which time is nested within each twin and each twin is nested within a twin pair. To conduct an analysis such as this, however, one would need access to a data source that includes a much larger sample and multiple repeated measures to pin down the timing of justice system contact and delinquency (something that is currently not available in the E‐Risk).

Third, the consistent pattern of labeling effects across our models lends confidence that these average effects are indeed real. Therefore, future studies should be aimed at dissecting the mediating mechanisms that cause justice system contact to promote delinquency. Although many researchers have explored how justice system contact indirectly increases criminal behavior through certain structural impediments that restrict life chances (e.g., Bernburg & Krohn, 2003; Chiricos et al., 2007; Lopes et al., 2012; Pager, 2003), we believe there is room to explore mediating variables that capture how justice system contact impacts an adolescent's identity. For instance, does justice system contact encourage adolescents to generate a “condemnation script” through which they begin to feel pessimism for their future and perceive themselves as being “doomed to deviance” (Maruna, 2001, p. 74)? We encourage future scholarship to explore the mechanisms that could be conduits for labeling outcomes (e.g., anger [Agnew, 1992], stigmatizing shame [Braithwaite, 1989], and sense of injustice [Sherman, 1993]).

### 4.3 Broader considerations

With the contributions of this study in mind, we now consider the broader substantive, theoretical, and ethical concerns that may stem from them. Particularly, we focus on the concerns revolving around the role of the justice system and its impact on juveniles. With evidence that the impact of contact with the justice system is a substantively negative one, an interesting question can be raised: Why would we have expected contact with the justice system to have a deterrent effect? Perhaps if justice system contact caused people to “fear their future self,” we would see deterrent effects (Paternoster & Bushway, 2009). But what we found is that justice system contact may have the opposite effect—rather than causing people to fear their future self, it may cause them to lose confidence in their future self. Therefore, the current system may work in a way that does not motivate individuals to conform to the norms of society. Instead, it leads young people to doubt their ability to get themselves out of the hole they have dug.

This makes sense when we consider the real‐life consequences of spending time in jail—the event itself is often embarrassing and shameful. Typically, it consists of (at least) an overnight stay followed by a visit with a judge the next morning. The family often has to get involved for the young person to be released back into the community, which then causes anger, hostility, and embarrassment among family members. Given that family is an important part of the desistance process, weakening those social bonds is unlikely to have a crime‐reducing effect. Furthermore, these reactions are often extended out to other interpersonal relationships in different settings, and as these relationships are ruined, prosocial connections are further attenuated, pushing the labeled adolescent further away from conventional society.

What does this mean for the justice system as it is currently constructed? We do not believe our findings show support for a shift to nonintervention. Rather, we believe it is important for the justice system and its actors to recognize the potentially negative impact it has. The public should be aware that the system is for the purpose of justice and retribution and that a utilitarian outcome such as specific deterrence is unlikely. With this in mind, our findings can be used to extend two policy recommendations.

First, although not a test of these hypotheses, we believe our findings fall in line with the arguments of the principles of effective intervention (see Andrews, 1995; Bonta & Andrews, 2016; Gendreau, 1996), which propose low‐risk offenders should not be funneled through official justice system channels. There should be diversionary programs set up for these types of offenders so that they may be able to avoid the labeling process. A metaphor might help explain: Medical doctors do not send a patient suffering from a cold to the emergency room. Even though the patient can certainly get treatment there, the visit would likely be counterproductive as the patient would be exposed to far more harmful viruses and diseases that may ultimately result in worse health. Study findings have repeatedly shown that when low‐risk offenders are brought into the justice system, the outcome is almost exclusively iatrogenic (e.g., Gatti, Tremblay, & Vitaro, 2009; Lowenkamp, Latessa, & Holsinger, 2006; Nagin, Cullen, & Jonson, 2009; Sperber, Latessa, & Makarios, 2013).

Second, our findings do not show support for fewer (or more) juvenile arrests. But they do indicate that if arrest rates are going to be maintained at their current level (or if they are to be heightened), then there should be a concerted effort toward offsetting the negative pathways that they create. If policy makers gain an understanding of these processes and pathways, they can develop and implement strategies to prevent labeling effects. Only then will the system have a chance of deterring criminal activity by way of contact with the offender.

## Supporting information

Figure S1. Histogram of the Distribution of Index of Multiple Deprivations (IMD) for the E‐Risk Cohort Compared With the Distribution of Deprivations in the United Kingdom (*N* = 2,232)
Table S1. Regression of Illegal Delinquency at Age 18 on Spent Night in Jail/Prison and Covariates
Table S2. Regression of Illegal Delinquency at Age 18 on Being Issued an Anti‐social Behaviour Order (ASBO) and Covariates
Table S3. Regression of Illegal Delinquency at Age 18 on Having an Official Crime Record Before Age 17 and Covariates
Table S4. Poisson Regression of Delinquency at Age 18 on Spent Night in Jail/Prison and Covariates
Table S5. Poisson Regression of Delinquency at Age 18 on Being Issued an Anti‐social Behaviour Order (ASBO) and Covariates
Table S6. Poisson Regression of Delinquency at Age 18 on Having an Official Crime Record Before Age 17 and Covariates
Table S7. Poisson Regression of Illegal Delinquency at Age 18 on Spent Night in Jail/Prison and Covariates
Table S8. Poisson Regression of Illegal Delinquency at Age 18 on Being Issued an Anti‐social Behaviour Order (ASBO) and Covariates
Table S9. Poisson Regression of Illegal Delinquency at Age 18 on Having an Official Crime Record Before Age 17 and Covariates

## APPENDIX A DESCRIPTION OF VARIABLES AND SCALES

### A.1

**Table:**

Variable/Scale	Informant	Description of Measure
Delinquency at age 18	Participant	During data collection, self‐reported conduct problems and delinquent offense behaviors were assessed through a computer questionnaire. The monitor was positioned so that the twin saw the questions in private and twins wore headphones to hear each question asked aloud. Participants self‐reported about their behavior in the past 12 months. All items were specifically selected to map onto the *DSM‐IV* criteria for conduct disorder (APA, 1994) and included fighting, bullying, being cruel to others, being cruel to animals, using weapons, vandalism, lying, robbery, shoplifting, breaking into homes or cars to steal, running away, and truancy. Available responses included “yes” and “no”. To create the scale, “yes” responses were coded to equal 1 and responses to each item were combined by summing across the 13 items. This generated a conduct problems and delinquent offenses scale with a possible range of 0 to 13.
Jail/prison	Participant	During the age‐18 interview, each participant was asked “Have you ever had to spend a night in police custody, jail, or prison?” while completing a computerized questionnaire that was positioned so all responses were completed in private. Responses were coded such that *no *= 0 and *yes* = 1. The reporting period was “ever” from age 10 up to age 18.
ASBO	Participant	During the age‐18 interview, each participant was asked “Have you ever been issued an ASBO (Anti‐social Behaviour Order)?” while completing a computerized questionnaire that was positioned so all responses were completed in private. Responses were coded such that *no *= 0 and *yes* = 1. The reporting period was “ever” from age 10 up to age 18. The earliest ASBO that was issued was at age 10.
Crime record	United Kingdom Ministry of Justice	Official records of participants’ cautions and convictions were obtained through United Kingdom Police National Computer (PNC) record searches conducted in cooperation with the United Kingdom Ministry of Justice. Records include complete histories of cautions and convictions for participants cautioned or convicted in the United Kingdom beginning at age 10 years (age of criminal responsibility). At the time of the current study, data were complete through age 19 years. Cautions and convictions were recoded into a binary variable to reflect whether participants had been cautioned or convicted or not. The variable was then further restricted to reflect whether a participant had received a caution or conviction before the age 17 years. Responses were coded such that *no *= 0 and *yes* = 1.
Delinquency at age 12	Participant	A computerized questionnaire was used to obtain self‐reports of antisocial behaviors when the twins were 12 years old. All items were specifically selected to map onto the *DSM‐IV* criteria for conduct disorder (APA, 1994). Items included the use of weapons (e.g., “Have you ever used a weapon on someone like a knife, piece of wood or baseball bat?”), truancy (e.g., “Do you sometimes skip school when you shouldn't?”), and stealing (e.g., “Have you stolen something while nobody was looking?”). Computer monitors were positioned so that the twin saw the questions in private, and twins wore headphones to hear each question aloud. Children responded with “yes” or “no” and were given the opportunity to refuse to answer. Scores were summed to create a single score indexing antisocial behaviors at age 12, with higher scores indicating greater involvement in antisocial behaviors (range = 0 to 24).
Externalizing problems at age 12	Mother, teacher	Children's externalizing problems were assessed with the Child Behavior Checklist (Achenbach, 1991a) and the Teacher's Report Form (Achenbach, 1991b), the most widely used and well‐validated assessment scheme for assessing antisocial behavior problems among children and adolescents. Mother interviews and teacher reports were combined by summing the items from each rater (scored 0–2); the internal consistency reliabilities of the parents’ and teachers’ reports were .89 and .94, respectively.
Low self‐control	Mother, teacher, and participant	Children's self‐control during their first decade of life was measured using a multi‐occasion/multi‐informant strategy, following Moffitt et al. (2011). Briefly, a self‐control factor was estimated via nine measures, including observational ratings of participants’ lack of control (age 5 years), parent and teacher reports of poor impulse control (ages 5, 7, and 10 years), participants’ self‐reports of inattentive and impulsive behavior (age 7 years), and interviewer judgments of the personality trait of Conscientiousness (age 10 years). On the basis of principal component analysis, we averaged the standardized measures into a single composite score with a mean of 0 and a standard deviation of 1.
Cognitive ability at age 12	Participant	Participants were tested using a short form of the Wechsler Intelligence Scale for Children, Fourth Edition (2003). Scores were standardized to have a mean of 100 and a standard deviation of 15.
Educational achievement	Participant	At age 18, participants were classified into four groups according to the highest educational qualification they attained according to their General Certificate of Secondary Education (GCSEs; a standardized examination taken at the end of compulsory education at 16 years of age. Groups included: *leaving school with no qualification* ( = 0); *leaving school with low‐average grades on GCSEs* ( = 1); *leaving school with grades A‐C on their GCSEs* ( = 2); or *sitting for Advanced level qualifications (known as A levels)* ( = 3). By age 18, 22% of the cohort left school with no qualification or only a GCSE at grades D‐G; at the other end, 50% of the cohort sat A levels.
Twin identifier	Parents	Parents were asked to indicate which twin in the twin‐pair was first born. Responses were coded such that the *elder twin *= 1 and the *younger twin* = 0.
Zygosity	Mother	Zygosity was determined using a standard zygosity questionnaire that has been shown to have 95% accuracy (Price et al., 2000) and subsequently confirmed when obtaining genome‐wide single nucleotide polymorphism (SNP) data.
