# Supplementary material for: Does contact with the justice system deter or promote future delinquency? Results from a longitudinal study of British adolescent twins
Source: Criminology. 2019 Dec 29;58(2):307–35. doi: 10.1111/1745-9125.12236 (PMC7317788; doi:10.1111/1745-9125.12236)
Supplement: Supplementary file 1 — Figure S1. Histogram of the Distribution of Index of Multiple Deprivations (IMD) for the E‐Risk Cohort Compared With the Distribution of Deprivations in the United Kingdom (N = 2,232) Table S1. Regression of Illegal Delinquency at Age 18 on Spent Night in Jail/Prison and Covariates Table S2. Regression of Illegal Delinquency at Age 18 on Being Issued an Anti‐social Behaviour Order (ASBO) and Covariates Table S3. Regression of Illegal Delinquency at Age 18 on Having an Official Crime Record Before Age 17 and Covariates Table S4. Poisson Regression of Delinquency at Age 18 on Spent Night in Jail/Prison and Covariates Table S5. Poisson Regression of Delinquency at Age 18 on Being Issued an Anti‐social Behaviour Order (ASBO) and Covariates Table S6. Poisson Regression of Delinquency at Age 18 on Having an Official Crime Record Before Age 17 and Covariates Table S7. Poisson Regression of Illegal Delinquency at Age 18 on Spent Night in Jail/Prison and Covariates Table S8. Poisson Regression of Illegal Delinquency at Age 18 on Being Issued an Anti‐social Behaviour Order (ASBO) and Covariates Table S9. Poisson Regression of Illegal Delinquency at Age 18 on Having an Official Crime Record Before Age 17 and Covariates [file CRIM-58-307-s001.docx]

SUPPORTING INFORMATION FOR

“DOES CONTACT WITH THE JUSTICE SYSTEM DETER OR PROMOTE FUTURE DELINQUENCY? RESULTS FROM A LONGITUDINAL STUDY OF BRITISH ADOLESCENT TWINS”^[[1]](#footnote-1)^*

RYAN T. MOTZ, J.C. BARNES, AVSHALOM CASPI, LOUISE ARSENEAULT, FRANCIS T. CULLEN, RENATE HOUTS, JASMIN WERTZ, and TERRIE E. MOFFITT

| **Figure S1. Histogram of the Distribution of Index of Multiple Deprivations (IMD) for the E-Risk Cohort Compared With the Distribution of Deprivations in the United Kingdom (*N* = 2,232)** |
| --- |

| *NOTES*: This histogram shows E-Risk families’ addresses are a near-perfect match to the deciles of the United Kingdom’s 2015 Lower-layer Super Output Area (LSOA) Index of Multiple Deprivation (IMD), which averages 1,500 residents; approximately 10% of the E-Risk cohort (dotted red line) fills each of the IMD’s 10% bands, indicating that the E-Risk cohort accurately represents the distribution of deprivation in the United Kingdom. |
| --- |

| **Table S1. Regression of Illegal Delinquency at Age 18 on Spent Night in Jail/Prison and Covariates** | | | | | | |
| --- | --- | --- | --- | --- | --- | --- |
|  | **No Fixed Effects**  **(MZ & DZ)** | | **Fixed Effects**  **(MZ & DZ)** | | **Fixed Effects**  **(MZ Only)** | |
| **Variables** | ***b***  **[95% CI]** | ***b***  **[95% CI]** | ***b***  **[95% CI]** | ***b***  **[95% CI]** | ***b***  **[95% CI]** | ***b***  **[95% CI]** |
| Key Independent Variable |  |  |  |  |  |  |
| Jail/prison = 1 | 1.954*** | 1.387*** | 1.213*** | 1.096*** | 1.038*** | 1.030*** |
|  | [1.628,2.280] | [1.051,1.723] | [.921,1.505] | [.803,1.388] | [.653,1.424] | [.647,1.412] |
| Covariates |  |  |  |  |  |  |
| Delinquency, age 12 | — | .099*** | — | .051*** | — | .050** |
|  |  | [.075,.124] |  | [.028,.074] |  | [.020,.081] |
| Ext. problems, age 12 | — | .006* | — | .001 | — | –.007 |
|  |  | [.001,.011] |  | [–.005,.007] |  | [–.016,.002] |
| Low self-control | — | .106*** | — | .076 | — | .217** |
|  |  | [.048,.164] |  | [–.011,.163] |  | [.067,.367] |
| Cognitive ability, age 12 | — | .007*** | — | .002 | — | .003 |
|  |  | [.004,.010] |  | [–.003,.008] |  | [–.005,.012] |
| Educational achievement | — | –.104** | — | –.110* | — | –.075 |
|  |  | [–.170,–.038] |  | [–.203,–.017] |  | [–.213,.064] |
| Twin identifier | — | –.075* | — | –.055 | — | –.041 |
|  |  | [–.144,–.006] |  | [–.121,.011] |  | [–.127,.045] |
| Family fixed effects  included? | No | | Yes | | Yes | |
| *N* | 1,901 | | 1,901 | | 1,068 | |

*NOTES:* The dependent variable (*illegal delinquency at age 18*) is a variety index of illegal behaviors that were self-reported at the age-18 interview. Specific offenses included fighting, using weapons, vandalism, robbery, breaking into homes or cars to steal, and shoplifting. Responses were coded *no* = 0 and *yes* = 1, which results in an index that ranged between 0 and 6 and had a mean of .639 with a standard deviation of 1.066.

*ABBREVIATIONS:* CI = confidence interval; DZ = dizygotic twin pairs; Ext. = externalizing; MZ = monozygotic twin pairs.

**p* < .05; ***p* < .01; ****p* < .001 (two-tailed).

**Table S2. Regression of Illegal Delinquency at Age 18 on Being Issued an Anti-social Behaviour Order (ASBO) and Covariates**

|  | **No Fixed Effects**  **(MZ & DZ)** | | **Fixed Effects**  **(MZ & DZ)** | | **Fixed Effects**  **(MZ Only)** | |
| --- | --- | --- | --- | --- | --- | --- |
| **Variables** | ***b***  **[95% CI]** | ***b***  **[95% CI]** | ***b***  **[95% CI]** | ***b***  **[95% CI]** | ***b***  **[95% CI]** | ***b***  **[95% CI]** |
| Key Independent Variable |  |  |  |  |  |  |
| ASBO = 1 | 2.169*** | 1.468*** | 1.600*** | 1.613*** | 2.000*** | 2.177*** |
|  | [1.573,2.766] | [.820,2.115] | [.953,2.247] | [.973,2.252] | [1.195,2.805] | [1.383,2.970] |
| Covariates |  |  |  |  |  |  |
| Delinquency, age 12 | — | .109*** | — | .057*** | — | .053*** |
|  |  | [.085,.134] |  | [.034,.081] |  | [.022,.083] |
| Ext. problems, age 12 | — | .009** | — | .002 | — | –.007 |
|  |  | [.003,.014] |  | [–.004,.008] |  | [–.016,.002] |
| Low self-control | — | .102*** | — | .065 | — | .234** |
|  |  | [.043,.162] |  | [–.024,.153] |  | [.084,.384] |
| Cognitive ability, age 12 | — | .008*** | — | .003 | — | .003 |
|  |  | [.005,.011] |  | [–.002,.009] |  | [–.006,.011] |
| Educational achievement | — | –.159*** | — | –.155** | — | –.127 |
|  |  | [–.230,–.089] |  | [–.249,–.061] |  | [–.265,.010] |
| Twin identifier | — | –.081* | — | –.061 | — | –.052 |
|  |  | [–.151,–.012] |  | [–.128,.006] |  | [–.138,.034] |
| Family fixed effects  included? | No | | Yes | | Yes | |
| *N* | 1,901 | | 1,901 | | 1,068 | |

*NOTES:* The dependent variable (*illegal delinquency at age 18*) is a variety index of illegal behaviors that were self-reported at the age-18 interview. Specific offenses included fighting, using weapons, vandalism, robbery, breaking into homes or cars to steal, and shoplifting. Responses were coded *no* = 0 and *yes* = 1, which results in an index that ranged between 0 and 6 and had a mean of .639 with a standard deviation of 1.066.

*ABBREVIATIONS:* ASBO = anti-social behaviour order; CI = confidence interval; DZ = dizygotic twin pairs; Ext. = externalizing; MZ = monozygotic twin pairs.

**p*<.05; ***p*<.01; ****p*<.001 (two-tailed).

| **Table S3. Regression of Illegal Delinquency at Age 18 on Having an Official Crime Record Before Age 17 and Covariates** | | | | | | |
| --- | --- | --- | --- | --- | --- | --- |
|  | **No Fixed Effects**  **(MZ & DZ)** | | **Fixed Effects**  **(MZ & DZ)** | | **Fixed Effects**  **(MZ Only)** | |
| **Variables** | ***b***  **[95% CI]** | ***b***  **[95% CI]** | ***b***  **[95% CI]** | ***b***  **[95% CI]** | ***b***  **[95% CI]** | ***b***  **[95% CI]** |
| Key Independent Variable |  |  |  |  |  |  |
| Crime record = 1 | 1.266*** | .697*** | .617*** | .536*** | .552** | .596** |
|  | [.983,1.548] | [.407,.988] | [.352,.881] | [.274,.799] | [.180,.923] | [.223,.970] |
| Covariates |  |  |  |  |  |  |
| Delinquency, age 12 | — | .106*** | — | .049*** | — | .046** |
|  |  | [.081,.131] |  | [.026,.073] |  | [.015,.078] |
| Ext. problems, age 12 | — | .007** | — | .002 | — | –.007 |
|  |  | [.002,.012] |  | [–.004,.008] |  | [–.016,.002] |
| Low self-control | — | .109*** | — | .081 | — | .215** |
|  |  | [.049,.168] |  | [–.007,.170] |  | [.063,.368] |
| Cognitive ability, age 12 | — | .008*** | — | .004 | — | .005 |
|  |  | [.005,.011] |  | [–.002,.009] |  | [–.004,.013] |
| Educational achievement | — | –.144*** | — | –.157** | — | –.153* |
|  |  | [–.215,–.073] |  | [–.252,–.063] |  | [–.295,–.011] |
| Twin identifier | — | –.071* | — | –.051 | — | –.027 |
|  |  | [–.141,–.001] |  | [–.118,.016] |  | [–.115,.060] |
| Family fixed effects  included? | No | | Yes | | Yes | |
| *N* | 1,901 | | 1,901 | | 1,068 | |

*NOTES:* The dependent variable (*illegal delinquency at age 18*) is a variety index of illegal behaviors that were self-reported at the age-18 interview. Specific offenses included fighting, using weapons, vandalism, robbery, breaking into homes or cars to steal, and shoplifting. Responses were coded *no* = 0 and *yes* = 1, which results in an index that ranged between 0 and 6 and had a mean of .639 with a standard deviation of 1.066.

*ABBREVIATIONS:* CI = confidence interval; DZ = dizygotic twin pairs; Ext. = externalizing; MZ = monozygotic twin pairs.

**p* < .05; ***p* < .01; ****p* < .001 (two-tailed).

| **Table S4. Poisson Regression of Delinquency at Age 18 on Spent Night in Jail/Prison and Covariates** | | | | | | |
| --- | --- | --- | --- | --- | --- | --- |
|  | **No Fixed Effects**  **(MZ & DZ)** | | **Fixed Effects**  **(MZ & DZ)** | | **Fixed Effects**  **(MZ Only)** | |
| **Variables** | ***b***  **[95% CI]** | ***b***  **[95% CI]** | ***b***  **[95% CI]** | ***b***  **[95% CI]** | ***b***  **[95% CI]** | ***b***  **[95% CI]** |
| Key Independent Variable |  |  |  |  |  |  |
| Jail/prison = 1 | .911*** | .525*** | .497*** | .395*** | .413** | .373** |
|  | [.815,1.007] | [.415,.634] | [.291,.703] | [.181,.608] | [.149,.676] | [.099,.646] |
| Covariates |  |  |  |  |  |  |
| Delinquency, age 12 | — | .063*** | — | .053*** | — | .049*** |
|  |  | [.053,.073] |  | [.033,.073] |  | [.022,.077] |
| Ext. problems, age 12 | — | .005*** | — | .006* | — | .002 |
|  |  | [.002,.008] |  | [.000,.011] |  | [–.006,.010] |
| Low self-control | — | .101*** | — | .052 | — | .190* |
|  |  | [.059,.144] |  | [–.034,.138] |  | [.031,.349] |
| Cognitive ability, age 12 | — | .006*** | — | .004 | — | .003 |
|  |  | [.003,.008] |  | [–.002,.009] |  | [–.006,.012] |
| Educational achievement | — | –.040 | — | –.031 | — | –.030 |
|  |  | [–.087,.007] |  | [–.116,.054] |  | [–.166,.106] |
| Twin identifier | — | –.050 | — | –.043 | — | –.039 |
|  |  | [–.115,.016] |  | [–.109,.024] |  | [–.129,.050] |
| Family fixed effects  included? | No | | Yes | | Yes | |
| *N* | 1,494 | | 1,494 | | 818 | |

*NOTES: n* = 308 cases omitted from the analysis due to both twins scoring 0 on the outcome;

*ABBREVIATIONS:* CI = confidence interval; DZ = dizygotic twin pairs; Ext. = externalizing; MZ = monozygotic twin pairs.

**p* < .05; ***p* < .01; ****p* < .001 (two-tailed).

**Table S5. Poisson Regression of Delinquency at Age 18 on Being Issued an Anti-social Behaviour Order (ASBO) and Covariates**

|  | **No Fixed Effects**  **(MZ & DZ)** | | **Fixed Effects**  **(MZ & DZ)** | | **Fixed Effects**  **(MZ Only)** | |
| --- | --- | --- | --- | --- | --- | --- |
| **Variables** | ***b***  **[95% CI]** | ***b***  **[95% CI]** | ***b***  **[95% CI]** | ***b***  **[95% CI]** | ***b***  **[95% CI]** | ***b***  **[95% CI]** |
| Key Independent Variable |  |  |  |  |  |  |
| ASBO = 1 | .907*** | .530*** | .934*** | .989*** | 1.099** | 1.330*** |
|  | [.734,1.080] | [.350,.710] | [.441,1.427] | [.478,1.500] | [.416,1.781] | [.636,2.025] |
| Covariates |  |  |  |  |  |  |
| Delinquency, age 12 | — | .069*** | — | .058*** | — | .052*** |
|  |  | [.059,.079] |  | [.038,.078] |  | [.024,.080] |
| Ext. problems, age 12 | — | .006*** | — | .005 | — | .002 |
|  |  | [.004,.009] |  | [–.000,.010] |  | [–.005,.010] |
| Low self-control | — | .101*** | — | .049 | — | .219** |
|  |  | [.058,.143] |  | [–.038,.135] |  | [.060,.379] |
| Cognitive ability, age 12 | — | .006*** | — | .004 | — | .002 |
|  |  | [.003,.009] |  | [–.001,.010] |  | [–.007,.011] |
| Educational achievement | — | –.077*** | — | –.071 | — | –.067 |
|  |  | [–.122,–.031] |  | [–.155,.013] |  | [–.202,.069] |
| Twin identifier | — | –.057 | — | –.056 | — | –.052 |
|  |  | [–.122,.009] |  | [–.123,.010] |  | [–.142,.037] |
| Family fixed effects  included? | No | | Yes | | Yes | |
| *N* | 1,494 | | 1,494 | | 818 | |

*NOTES: n* = 308 cases omitted from the analysis due to both twins scoring 0 on the outcome.

*ABBREVIATIONS:* ASBO = anti-social behaviour order; CI = confidence interval; DZ = dizygotic twin pairs; Ext. = externalizing; MZ = monozygotic twin pairs.

**p* < .05; ***p* < .01; ****p* < .001 (two-tailed).

| **Table S6. Poisson Regression of Delinquency at Age 18 on Having an Official Crime Record Before Age 17 and Covariates** | | | | | | |
| --- | --- | --- | --- | --- | --- | --- |
|  | **No Fixed Effects**  **(MZ & DZ)** | | **Fixed Effects**  **(MZ & DZ)** | | **Fixed Effects**  **(MZ Only)** | |
| **Variables** | ***b***  **[95% CI]** | ***b***  **[95% CI]** | ***b***  **[95% CI]** | ***b***  **[95% CI]** | ***b***  **[95% CI]** | ***b***  **[95% CI]** |
| Key Independent Variable |  |  |  |  |  |  |
| Crime record = 1 | .650*** | .253*** | .309** | .262* | .236 | .274 |
|  | [.555,.745] | [.145,.362] | [.108,.509] | [.057,.466] | [–.040,.513] | [–.016,.563] |
| Covariates |  |  |  |  |  |  |
| Delinquency, age 12 | — | .066*** | — | .052*** | — | .048*** |
|  |  | [.056,.076] |  | [.032,.072] |  | [.021,.076] |
| Ext. problems, age 12 | — | .005*** | — | .006* | — | .003 |
|  |  | [.003,.008] |  | [.001,.011] |  | [–.005,.010] |
| Low self-control | — | .109*** | — | .059 | — | .195* |
|  |  | [.067,.152] |  | [–.027,.145] |  | [.036,.354] |
| Cognitive ability, age 12 | — | .006*** | — | .004 | — | .003 |
|  |  | [.003,.009] |  | [–.001,.010] |  | [–.006,.012] |
| Educational achievement | — | –.067** | — | –.061 | — | –.079 |
|  |  | [–.113,–.020] |  | [–.145,.022] |  | [–.217,.059] |
| Twin identifier | — | –.048 | — | –.042 | — | –.035 |
|  |  | [–.114,.017] |  | [–.108,.024] |  | [–.124,.054] |
| Family fixed effects  included? | No | | Yes | | Yes | |
| *N* | 1,494 | | 1,494 | | 818 | |

*NOTES: n* = 308 cases omitted from the analysis due to both twins scoring 0 on the outcome.

*ABBREVIATIONS:* CI = confidence interval; DZ = dizygotic twin pairs; Ext. = externalizing; MZ = monozygotic twin pairs.

**p* < .05; ***p* < .01; ****p* < .001 (two-tailed).

| **Table S7. Poisson Regression of Illegal Delinquency at Age 18 on Spent Night in Jail/Prison and Covariates** | | | | | | |
| --- | --- | --- | --- | --- | --- | --- |
|  | **No Fixed Effects**  **(MZ & DZ)** | | **Fixed Effects**  **(MZ & DZ)** | | **Fixed Effects**  **(MZ Only)** | |
| **Variables** | ***b***  **[95% CI]** | ***b***  **[95% CI]** | ***b***  **[95% CI]** | ***b***  **[95% CI]** | ***b***  **[95% CI]** | ***b***  **[95% CI]** |
| Key Independent Variable |  |  |  |  |  |  |
| Jail/prison = 1 | .838*** | .599*** | .721*** | .595*** | .537** | .526* |
|  | [.690,.986] | [.429,.769] | [.395,1.046] | [.255,.936] | [.137,.937] | [.110,.943] |
| Covariates |  |  |  |  |  |  |
| Delinquency, age 12 | — | .047*** | — | .052** | — | .037 |
|  |  | [.029,.064] |  | [.017,.086] |  | [–.010,.084] |
| Ext. problems, age 12 | — | –.000 | — | .001 | — | –.008 |
|  |  | [–.005,.004] |  | [–.008,.009] |  | [–.021,.005] |
| Low self-control | — | .114** | — | .115 | — | .267 |
|  |  | [.037,.192] |  | [–.038,.268] |  | [–.009,.543] |
| Cognitive ability, age 12 | — | .004 | — | .005 | — | .006 |
|  |  | [–.001,.009] |  | [–.005,.015] |  | [–.011,.022] |
| Educational achievement | — | –.069 | — | –.122 | — | –.107 |
|  |  | [–.152,.015] |  | [–.273,.028] |  | [–.341,.127] |
| Twin identifier | — | –.087 | — | –.082 | — | –.060 |
|  |  | [–.206,.032] |  | [–.203,.039] |  | [–.219,.099] |
| Family fixed effects  included? | No | | Yes | | Yes | |
| *N* | 860 | | 860 | | 464 | |

*NOTES:* The dependent variable (*illegal delinquency at age 18*) is a variety index of illegal behaviors that were self-reported at the age-18 interview. Specific offenses included fighting, using weapons, vandalism, robbery, breaking into homes or cars to steal, and shoplifting. Responses were coded *no* = 0 and *yes* = 1, which results in an index that ranged between 0 and 6 and had a mean of .639 with a standard deviation of 1.066. *n* = 936 cases omitted from the analysis due to both twins scoring 0 on the outcome.

*ABBREVIATIONS:* CI = confidence interval; DZ = dizygotic twin pairs; Ext. = externalizing; MZ = monozygotic twin pairs.

**p* < .05; ***p* < .01; ****p* < .001 (two-tailed).

**Table S8. Poisson Regression of Illegal Delinquency at Age 18 on Being Issued an Anti-social Behaviour Order (ASBO) and Covariates**

|  | **No Fixed Effects**  **(MZ & DZ)** | | **Fixed Effects**  **(MZ & DZ)** | | **Fixed Effects**  **(MZ Only)** | |
| --- | --- | --- | --- | --- | --- | --- |
| **Variables** | ***b***  **[95% CI]** | ***b***  **[95% CI]** | ***b***  **[95% CI]** | ***b***  **[95% CI]** | ***b***  **[95% CI]** | ***b***  **[95% CI]** |
| Key Independent Variable |  |  |  |  |  |  |
| ASBO = 1 | .757*** | .503*** | 1.190** | 1.440** | 1.224* | 1.561** |
|  | [.493,1.022] | [.228,.777] | [.344,2.036] | [.568,2.313] | [.227,2.221] | [.534,2.588] |
| Covariates |  |  |  |  |  |  |
| Delinquency, age 12 | — | .053*** | — | .061*** | — | .041 |
|  |  | [.036,.071] |  | [.026,.096] |  | [–.006,.089] |
| Ext. problems, age 12 | — | .001 | — | .001 | — | –.007 |
|  |  | [–.003,.006] |  | [–.008,.009] |  | [–.020,.006] |
| Low self-control | — | .117** | — | .123 | — | .321* |
|  |  | [.040,.194] |  | [–.031,.277] |  | [.044,.598] |
| Cognitive ability, age 12 | — | .004 | — | .005 | — | .003 |
|  |  | [–.000,.009] |  | [–.005,.015] |  | [–.013,.020] |
| Educational achievement | — | –.126** | — | –.190* | — | –.167 |
|  |  | [–.207,–.044] |  | [–.337,–.043] |  | [–.401,.068] |
| Twin identifier | — | –.084 | — | –.098 | — | –.084 |
|  |  | [–.203,.035] |  | [–.220,.023] |  | [–.245,.076] |
| Family fixed effects  included? | No | | Yes | | Yes | |
| *N* | 860 | | 860 | | 464 | |

*NOTES:* The dependent variable (*illegal delinquency at age 18*) is a variety index of illegal behaviors that were self-reported at the age-18 interview. Specific offenses included fighting, using weapons, vandalism, robbery, breaking into homes or cars to steal, and shoplifting. Responses were coded *no* = 0 and *yes* = 1, which results in an index that ranged between 0 and 6 and had a mean of .639 with a standard deviation of 1.066. *n* = 936 cases omitted from the analysis due to both twins scoring 0 on the outcome.

*ABBREVIATIONS:* CI = confidence interval; DZ = dizygotic twin pairs; Ext. = externalizing; MZ = monozygotic twin pairs.

**p* < .05; ***p* < .01; ****p* < .001 (two-tailed).

| **Table S9. Poisson Regression of Illegal Delinquency at Age 18 on Having an Official Crime Record Before Age 17 and Covariates** | | | | | | |
| --- | --- | --- | --- | --- | --- | --- |
|  | **No Fixed Effects**  **(MZ & DZ)** | | **Fixed Effects**  **(MZ & DZ)** | | **Fixed Effects**  **(MZ Only)** | |
| **Variables** | ***b***  **[95% CI]** | ***b***  **[95% CI]** | ***b***  **[95% CI]** | ***b***  **[95% CI]** | ***b***  **[95% CI]** | ***b***  **[95% CI]** |
| Key Independent Variable |  |  |  |  |  |  |
| Crime record = 1 | .564*** | .295*** | .487** | .469** | .359 | .524* |
|  | [.414,.714] | [.122,.468] | [.163,.811] | [.135,.803] | [–.061,.779] | [.078,.971] |
| Covariates |  |  |  |  |  |  |
| Delinquency, age 12 | — | .050*** | — | .050** | — | .035 |
|  |  | [.033,.068] |  | [.016,.084] |  | [–.012,.082] |
| Ext. problems, age 12 | — | .000 | — | .001 | — | –.008 |
|  |  | [–.005,.005] |  | [–.008,.010] |  | [–.021,.005] |
| Low self-control | — | .129*** | — | .135 | — | .291* |
|  |  | [.053,.206] |  | [–.018,.288] |  | [.015,.567] |
| Cognitive ability, age 12 | — | .005* | — | .005 | — | .007 |
|  |  | [.000,.010] |  | [–.005,.016] |  | [–.009,.024] |
| Educational achievement | — | –.111** | — | –.188* | — | –.212 |
|  |  | [–.193,–.028] |  | [–.336,–.041] |  | [–.451,.028] |
| Twin identifier | — | –.074 | — | –.080 | — | –.055 |
|  |  | [–.193,.045] |  | [–.201,.041] |  | [–.214,.104] |
| Family fixed effects  included? | No | | Yes | | Yes | |
| *N* | 860 | | 860 | | 464 | |

*NOTES:* The dependent variable (*illegal delinquency at age 18*) is a variety index of illegal behaviors that were self-reported at the age-18 interview. Specific offenses included fighting, using weapons, vandalism, robbery, breaking into homes or cars to steal, and shoplifting. Responses were coded *no* = 0 and *yes* = 1, which results in an index that ranged between 0 and 6 and had a mean of .639 with a standard deviation of 1.066. *n* = 936 cases omitted from the analysis due to both twins scoring 0 on the outcome.

*ABBREVIATIONS:* CI = confidence interval; DZ = dizygotic twin pairs; Ext. = externalizing; MZ = monozygotic twin pairs.

**p* < .05; ***p* < .01; ****p* < .001 (two-tailed).

1. *Published in *Criminology*, volume 58, issue 2, 2020. [↑](#footnote-ref-1)
